# Ocular microbiota types and longitudinal microbiota alterations in patients with chronic dacryocystitis with and without antibiotic pretreatment

**DOI:** 10.1002/imo2.17

**Published:** 2024-07-09

**Authors:** Shengru Wu, Limin Zhu, Tingting Wang, Chenguang Zhang, Jiaqi Lin, Yanjin He, Junhu Yao, Tingting Lin, Juan Du

**Affiliations:** ^1^ College of Animal Science and Technology Northwest A&F University Yangling China; ^2^ Department of Microbiology, Tumor and Cell Biology Karolinska Institute Solna Sweden; ^3^ Tianjin Key Laboratory of Retinal Functions and Diseases, Tianjin Branch of National Clinical Research Center for Ocular Disease, Eye Institute and School of Optometry Tianjin Medical University Eye Hospital Tianjin China; ^4^ Department of Ophthalmology of the First Hospital of Xi'an Shanxi Ophthalmological Institute Xi'an China

**Keywords:** antibiotic, chronic dacryocanaliculitis, chronic dacryocystitis, dacryocystorhinostomy, mucosa‐associated lymphoid tissue, ocular microbiota

## Abstract

Antibiotic pretreatment is routine for chronic dacryocystitis (DC) patients. Herein, the longitudinal effects of antibiotic pretreatment before dacryocystorhinostomy for DC patients were evaluated. Conjunctival and nasal swabs were collected longitudinally from 33 DC patients with and without antibiotic pretreatment, both before dacryocystorhinostomy and at 1, 2, and 4 weeks postdacryocystorhinostomy. Additionally, conjunctival sac swabs were collected from 46 healthy volunteers and 14 other ocular diseases patients. Comparisons focused on ocular/nasal microbiota and recovery outcomes. Compared to healthy participants, DC patients without antibiotic pretreatment exhibited greater ocular microbiota diversity before dacryocystorhinostomy. Although clinical recovery rates were comparable, our results suggest that, after antibiotic pretreatment, the ocular microbiota richness and diversity, and the composition alteration tendency, significantly changed 4 weeks after surgery. This implies that the ocular microbiota was more disturbed in patients who underwent antibiotic pretreatment compared to those without such treatment. Furthermore, two types of ocular microbiota and three types of nasal microbiota were identified in ocular diseases. This study provides comprehensive data on the ocular and nasal microbiota in DC patients with and without antibiotic pretreatment, along with other ocular diseases. This finding suggested that antibiotic pretreatment may not be necessary before dacryocystorhinostomy for DC patients, especially for nonsevere cases.

## Highlights

- Two types of ocular microbiota and three types of nasal microbiota were demonstrated among ocular diseases patients.
- Although clinical recovery is comparable, the antibiotic pretreatment significantly changed the ocular microbiota 4 weeks after dacryocystorhinostomy, which suggested the antibiotic is not needed for recovery from dacryocystorhinostomy.
- An altered ocular microbiota could potentially be used to assist in chronic dacryocystitis diagnosis.

## Graphical abstract

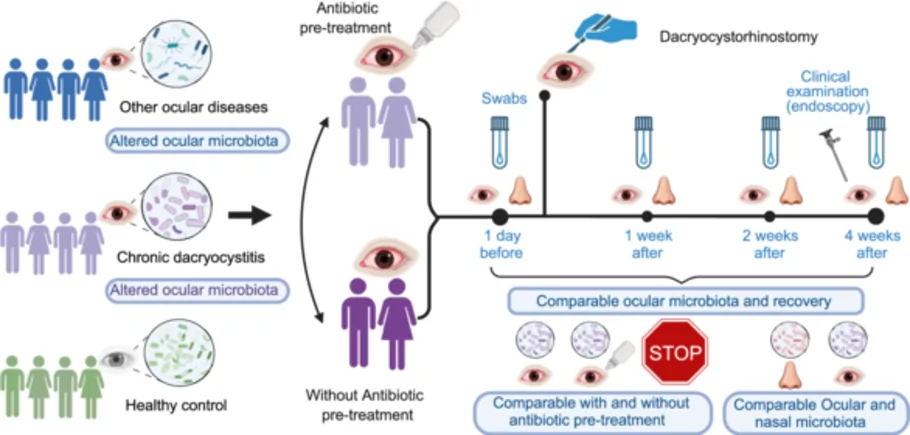

## 1 INTRODUCTION

As in other body sites, such as the gastrointestinal tract, skin, vaginal tract, and oral cavity [1, 2, 3, 4, 5], the human eye also contains a normal microbiota, which is expected to protect eye health from invaders [6, 7]. Increasing evidence indicates that an unbalanced microbiota is associated with the overgrowth of pathogenic microbes and local inflammation, which are linked to different ocular diseases [8, 9, 10, 11, 12, 13]. The ocular surface is directly exposed to the external environment and is endangered by various pathogenic microorganisms [14, 15, 16, 17, 18]. Both chronic dacryocystitis (DC) and chronic dacryocanaliculitis (DCC) are common chronic ocular inflammatory diseases [19, 20, 21]. Compared with DCC, stenosis of the lacrimal canaliculus (SLC) is an anomaly of an ocular anatomical structure that induces involuntary tearing without inflammation [22, 23, 24]. Furthermore, ocular mucosa‐associated lymphoid tissue (MALT) is clinically identified as a raised, fleshy, pink mass on the conjunctiva or as soft tissue under the conjunctiva [25, 26]. Few studies have indicated the association of ocular microbiota with the use of contact lenses and various ophthalmic diseases [9, 10, 11, 12, 13, 25, 27]. However, additional studies on ocular microbiota and related diseases, especially with longitudinal cohorts, will be highly valuable.

The DC is dominant among lacrimal drainage obstruction disorders. If not properly treated, DC can progress into severe ocular complications such as orbital cellulitis, abscess formation, and may even escalate to life‐threatening conditions like meningitis, cavernous sinus thrombosis, and so forth. Additionally, the prolonged presence of pathogenic bacteria associated with DC complicates the timing for intraocular surgeries, increasing the risk of infectious ocular surface diseases and infectious endophthalmitis [19, 20, 21]. Dacryocystorhinostomy is a surgery that creates a new path for tears to drain between the eyes and nose, assisting in reducing tear duct blockage [28, 29]. In China and many other countries, antibiotic pretreatment, especially levofloxacin pretreatment, is one of the most commonly applied antibiotics before dacryocystorhinostomy [30, 31, 32, 33]. There are hints about the effect of antibiotic use on the ocular microbiota, however, how this pretreatment benefit the recovery of the DC is understudied [8, 34, 35]. Antibiotic resistance is a growing global public health and environmental issue. Antibiotic abuse in clinical practice and animal feeding serve as the main contributors to antibiotic resistance [36]. Increasing antibiotic resistance among ocular pathogens is particularly worrisome [37, 38]. Hence, the necessity of these antibiotic prescriptions before dacryocystorhinostomy is worthy of thorough evaluation, considering the ocular microbiota recovery and overall improvement in clinical symptoms.

**Figure 1:**
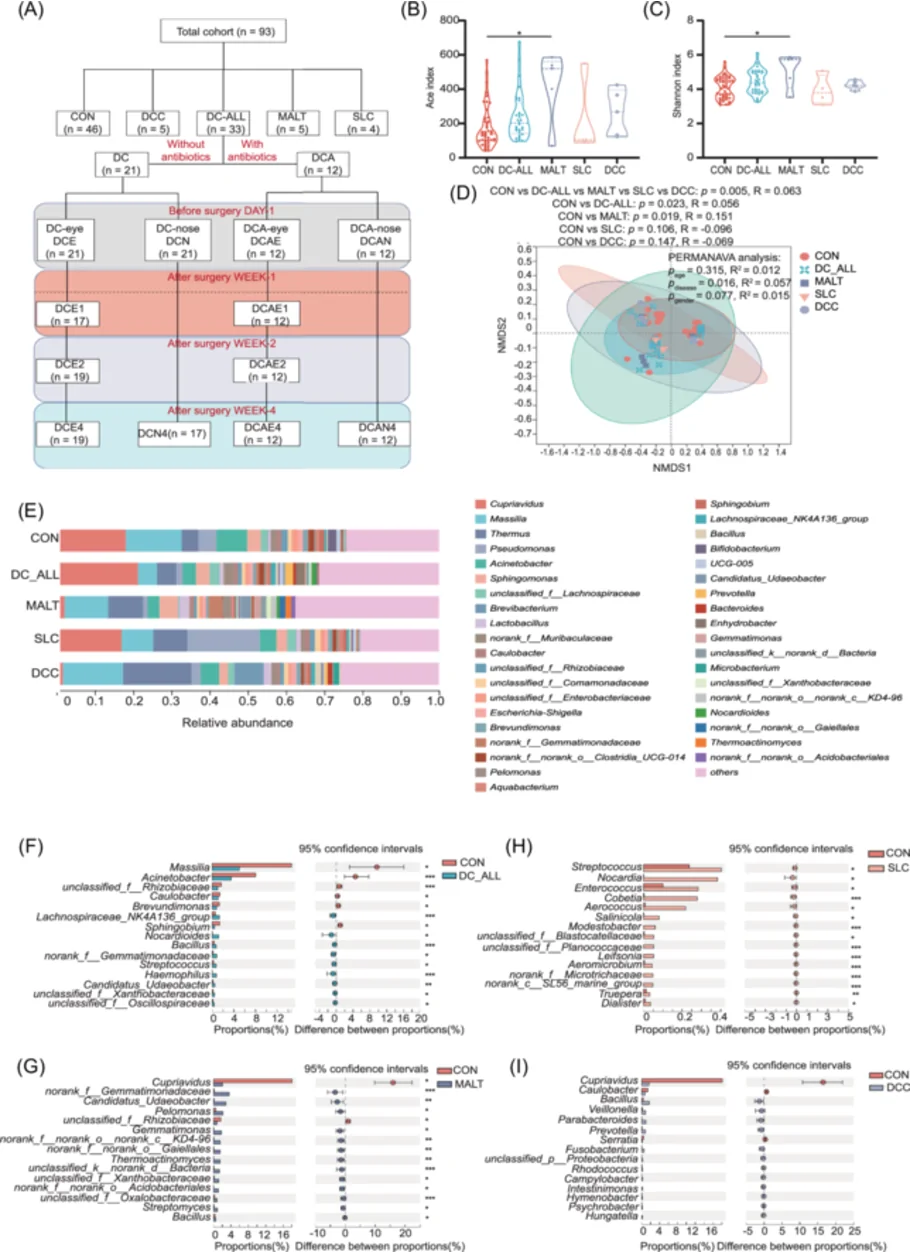
Flow chart for the study design and ocular microbiota in different ocular diseases. (A) The total cohort includes 46 healthy volunteers and 47 patients with 33 cases of chronic dacryocystitis (DC‐ALL), five cases of ocular mucosa‐associated lymphoid tissue (MALT), four cases of stenosis of lacrimal canaliculus (SLC), and five cases of chronic dacryocanaliculitis (DCC). Furthermore, 12 DC patients were pretreated with eye drops containing levofloxacin (DCAE), and 21 DC patients were not pretreated with antibiotics (DCE). Longitudinal samples were further collected to investigate the ocular (DCAE and DCE groups) and nasal microbiota (DCAN and DCN groups) during the dacryocystorhinostomy treatment and recovery over a 4‐week period. (B) Comparison of ocular microbial alpha diversity with Ace index among DC‐ALL, MALT, SLC, and DCC patients and healthy control group (CON). (C) Comparison of ocular microbial alpha diversity with Shannon index among DC‐ALL, MALT, SLC, and DCC patients and CON group. The Kruskal–Wallis test with Tukey–Kramer post hoc test was employed to test microbial alpha diversity differences. *FDR < 0.05. (D) Comparison of ocular microbial beta diversity with the ANOSIM analysis based on Bray–Curtis distance matric among the patient groups and CON group. In addition, the PERMANOVA analysis, considering age and gender effects, was applied. (E) The ocular microbial composition of the healthy participants, DC, MALT, SLC, and DCC patients. Only genera with relative abundance of more than 1% are listed. (F–I) Significantly different genera when comparing CON and DC‐ALL groups (F), CON and MALT groups (G), CON and SLC groups (H), CON and DCC groups (I). The Mann–Whitney *U* test was carried out for the two groups. *FDR < 0.05, **FDR < 0.01, ***FDR < 0.001.

Thus, we conducted a clinical cohort study on longitudinal samples of ocular and nasal microbiota obtained during DC dacryocystorhinostomy treatment and during recovery with or without antibiotic pretreatment. The data were also compared with those of healthy participants and patients with other eye diseases, including DCC, SLC, and MALT. In addition, the ocular and nasal microbiota changes were compared with the patients' clinical recovery records after dacryocystorhinostomy (Figure 1A).

## 2 RESULTS

### The ocular microbiota diversity of chronic DC and ocular MALT involving conjunctiva patients was significantly altered

We first compared the ocular microbiota diversity between healthy volunteers control group (CON) and patients with all the other ocular diseases included DC, DCC, SLC, and MALT (Figure 1A). Ocular microbiota samples from patients with DC collected before dacryocystorhinostomy were grouped into the DC‐ALL group and used for comparisons among healthy volunteers and patients with different ocular diseases. Only the patients in the MALT group exhibited significantly increased ocular microbiota diversity (Figure 1B,C, Figures S1A and S1B). The comparison of microbiota beta diversity revealed significant dissimilarity among all the diseases and control (*p*_ANOSIM_ = 0.005), especially for DC and MALT patients (*p*_ANOSIM_ = 0.023 and 0.019) (Figure 1D). Furthermore, according to the PERMANOVA results excluding potential interference from age and gender, significant dissimilarity in the microbiota was observed among all the groups (Figure 1D). *Cupriavidus*, *Massilia*, *Thermus*, *Pseudomonas*, and *Acinetobacter* were the top genera of all the groups, with various abundances in different diseases (Figure 1E). When comparing the proportions of each bacterial genus in healthy individuals, DC patients had significantly fewer *Massilia*, *Acinetobacter*, and *Rhizobiaceae* and more *Lachnospiraceae* NK4A136 group and *Nocardioides* (Figure 1F); MALT patients had significantly fewer *Cupriavidus* and *Rhizobiaceae* and more *Gemmatimonadaceae*, *Candidatus Udaeobacter*, and *Pelomonas* (Figure 1G); and SLC patients had more *Streptococcus*, *Nocardia*, *Enterococcus*, *Cobetia* and *Aerococcus* (Figure 1H). Moreover, the DCC patients exhibited significant decreases in *Cupriavidus* and *Caulobacter* and increases in *Bacillus*, *Veillonella*, *Parabacteroides*, and *Prevotella* (Figure 1I). This indicates different ocular surface diseases are associated with the specific ocular microbiota composition.

### Different ocular microbiotas were identified between healthy volunteers and DC patients

Due to the limited samples from MALT patients, we focused our study on DC patients, grouped them according to antibiotic pretreatment status, and monitored them for 4 weeks after dacryocystorhinostomy. All 12 DC patients in this study who underwent antibiotic pretreatment (DCA group) completed the 4‐week postoperative follow‐up medical examinations, and 19 out of the 21 patients without antibiotic pretreatment (DC group) completed the 4‐week follow‐up (Figure 1A). All patients underwent endoscopic dacryocystorhinostomy regardless of antibiotic pretreatment. Furthermore, no pathogen was detected by the conventional culturing method on the GC agar plates from all the DC patients regardless with and without antibiotic pretreatment.

We firstly compared the ocular microbiota of the control group with ocular microbiota from DC patients with and without antibiotic pretreatment (named as DCAE‐ALL and DCE‐ALL groups), by involving all conjunctival sac swab samples that collected 1 day before dacryocystorhinostomy, and at the first, second, and fourth weeks after dacryocystorhinostomy. Significant increases in microbial diversity (ACE) and microbial community richness (Shannon) were observed in DC patients without antibiotic pretreatment compared with those in the control group (Figures S2A and S2B). Furthermore, the comparison of microbiota beta diversity revealed significant dissimilarity (*p*_ANOSIM_ = 0.001 and *p*_Repeated measures aware PERMANOVA_ = 0.001) among the three groups (Figure S2C). Moreover, the differences between the control and DCE‐ALL groups (*p*_Repeated measures aware PERMANOVA_ = 0.001), between the control and DCAE‐ALL groups (*p*_Repeated measures aware PERMANOVA_ = 0.014), and between the DCAE‐ALL and DCE‐ALL groups were also detected (*p*_Repeated measures aware PERMANOVA_ = 0.006) (Figure S2C).

**Figure 2:**
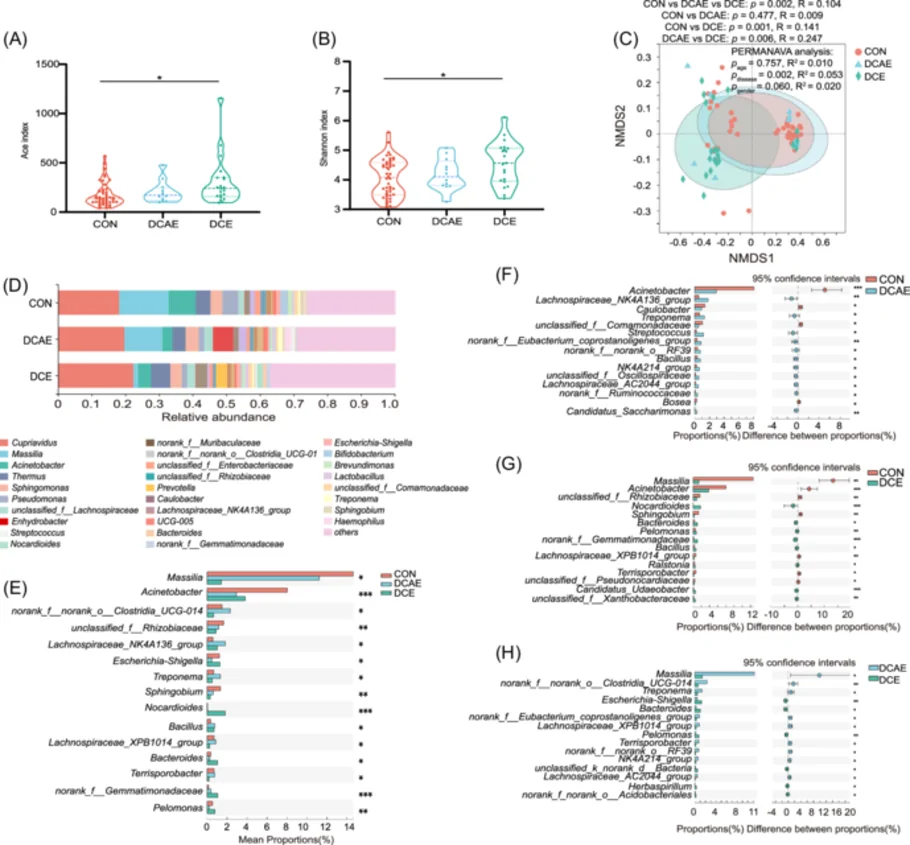
Comparison of ocular microbiota of dacryocystitis (DC) patients with (DCAE) and without (DCE) antibiotic pretreatment before the dacryocystorhinostomy. (A) Comparison of ocular microbial alpha diversity with Ace index among CON, DCAE, and DCE groups. (B) Comparison of ocular microbial alpha diversity with Shannon index among CON, DCAE, and DCE groups. The Kruskal–Wallis test with Tukey–Kramer post hoc test was employed to test microbial alpha diversity differences. *FDR < 0.05. (C) Comparison of ocular microbial beta diversity with the ANOSIM analysis based on Bray–Curtis distance matric among the CON, DCAE, and DCE groups. In addition, the PERMANOVA analysis considering age and gender effects was applied. (D) The microbial genera composition of the healthy participants, DCAE, and DCE patients. Only genera with relative abundance of more than 1% are listed. (E–H) Significantly different genera when comparing the CON, DCAE, and DCE groups (E), CON and DCAE groups (F), CON and DCE groups (G), DCAE and DCE groups (H). The Mann–Whitney *U* test was carried out for the two groups. *FDR < 0.05, **FDR < 0.01, ***FDR < 0.001.

Then, we compared the ocular microbiota from DC patients with and without antibiotic pretreatment (DCAE vs. DCE group), by only involving the samples collected before the dacryocystorhinostomy, with that of the control group. Similarly, significant increases in microbial diversity (ACE) and microbial community richness (Shannon) were observed in DC patients without antibiotic pretreatment than in the control group (Figure 2A,B Figure S1C–D). Furthermore, the comparison of microbiota beta diversity revealed significant dissimilarity (*p*_ANOSIM_ = 0.002 and *p*_PERMANOVA_ = 0.016) among the three groups (Figure 2C). Notable differences were detected between the control group and the DC patient group without antibiotic pretreatment (CON vs. DCE) and between the groups with and without antibiotic pretreatment (DCAE vs. DCE) (Figure 2C).

Among the 1847 genera identified in the ocular samples, the five main genera were *Cupriavidus*, *Massilia*, *Acinetobacter*, *Thermus*, and *Sphingomonas* (Figure 2D). A significant decrease in *Massillia*, *Acinetobacter*, *Rhizobiaceae*, and *Sphingobium* was noted in the DC group compared with the control group (Figure 2E). Furthermore, *Lachnospiraceae* NK4A136 group and *Treponema* were significantly increased in the DCAE group, and *Nocardioides*, *Bacteroides*, and *Gemmatimonadaceae* were significantly increased in the DCE group when compared with the other two groups (Figure 2E). When comparing each paired group, significant changes were observed in different bacterial species (Figure 2F–H).

### The ocular microbiota of DC patients was significantly altered by antibiotic pretreatment after dacryocystorhinostomy

**Figure 3:**
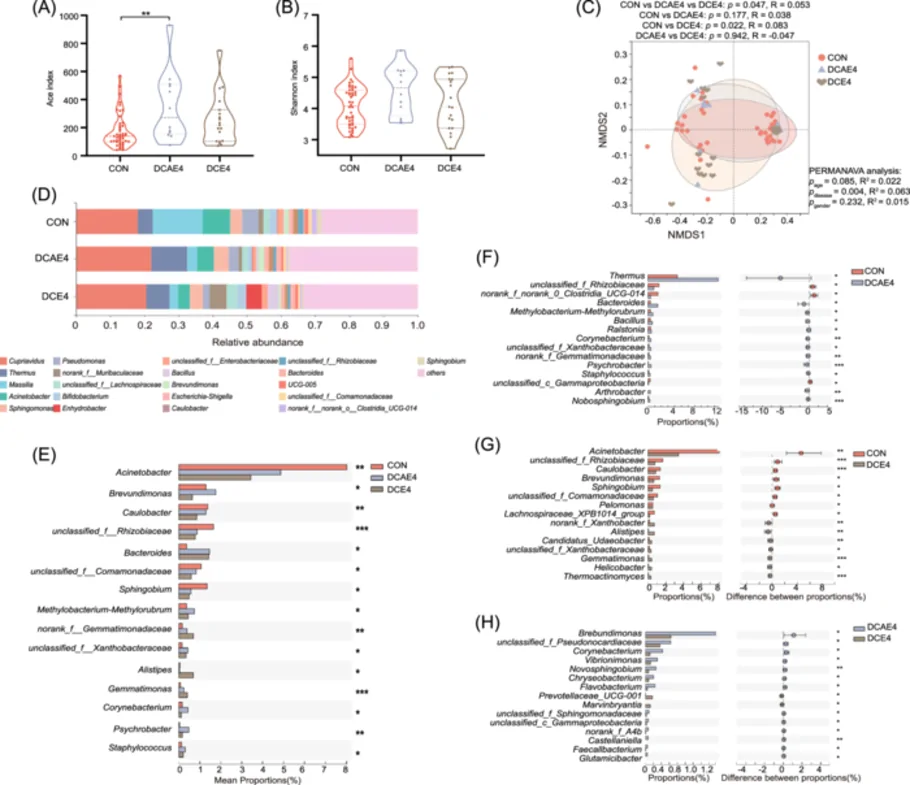
Comparison of ocular microbiota of dacryocystitis (DC) patients with (DCAE4) and without (DCE4) antibiotic pretreatment at the fourth week after the dacryocystorhinostomy. (A) Comparison of ocular microbial alpha diversity with Ace index among CON, DCAE4, and DCE4 groups. The Kruskal–Wallis test with Tukey–Kramer post hoc test was employed to test microbial alpha diversity differences. **FDR < 0.01. (B) Comparison of ocular microbial alpha diversity with Shannon index among CON, DCAE4, and DCE4 groups. (C) Comparison of ocular microbial beta diversity with the ANOSIM analysis based on Bray–Curtis distance matric among CON, DCAE4, and DCE4 groups. In addition, the PERMANOVA analysis considering age and gender effects was applied. (D) The microbial genera composition of the healthy participants, DCAE4, and DCE4 patients. Only genera with relative abundance of more than 1% are listed. (E–H) Significantly different genera when comparing the CON, DCAE4, and DCE4 groups (E), CON and DCAE4 groups (F), CON and DCE4 groups (G), DCAE4 and DCE4 groups (H). The Mann–Whitney *U* test was carried out for the two groups. *FDR < 0.05, **FDR < 0.01, ***FDR < 0.001.

We continued to monitor the ocular microbiota diversity till the last visit, the day for disease‐treatment evaluation (fourth week after dacryocystorhinostomy). When compared with the control group, significantly increased microbial community diversity (ACE) was observed in DC patients with antibiotic pretreatment (Figure 3A and Figure S1E–F). A similar trend was observed in the microbial community richness, although it did not reach the significant level (Figure 3B). Furthermore, the comparison of microbiota beta diversity presented a significant dissimilarity (*p*_ANOSIM_ = 0.047) among all three groups at the fourth week, especially when comparing the DC patient that without antibiotic pretreatment group with the control group (Figure 3C). Notably, the results hold by excluding the potential influence from age and gender using the PERMANOVA analysis (Figure 3C). Among the 1795 identified genera, the top five main genera were *Cupriavidus*, *Thermus*, *Massilia*, *Acinetobacter*, and *Sphingomonas* (Figure 3D). *Acinetobacter*, *Caulobacter*, *Rhizobiaceae*, *Comamonadaceae*, and *Sphingobium* were significantly decreased in DC groups (Figure 3E). *Brevundimonas*, *Bacteroides*, and *Methylobacterium*‐*Methylorubrum* were higher in the DCAE4 group, and *Gemmatimonadaceae* and *Alistipes* were higher in DCE4 group compared with the other two groups (Figure 3E). Compared with the control group, the patients in DCAE4 group exhibited a significantly increased *Thermus*, *Bacteroides*, and *Methylobacterium*‐*Methylorubrum* and decreased *Rhizobiaceae* and *Clostridia* UCG‐014 (Figure 3F). In addition, the patients in DCE4 group contained a significantly increased *Gemmatimonadaceae, Alistipes*, and *Candidatus Udaeobacter* and decreased *Acinetobacter*, *Rhizobiaceae*, and *Caulobacter* than the control group (Figure 3G). Furthermore, antibiotic pretreatment significantly increased the abundance of *Brevundimonas*, *Pseudonocardiaceae*, and *Corynebacterium* and significantly decreased *Prevotellaceae* UCG‐001 and *Marvinbryantia* by the fourth week after dacryocystorhinostomy (Figure 3H).

### Certain ocular bacteria assisted in distinguishing DC patients from healthy participants

To identify the potential microbial signatures for DC patients, we applied the random forest analysis for comparing healthy control group with both DCAE and DCE groups, as well as DCAE4 and DCE4 groups. To distinguish DCAE and DCE from the control group, top nine important genera with the lowest error rate of 0.327 (Figure S3A–B) are required. However, it needs 46 top important genera to obtain the lowest error rate of 0.361 to distinguish DCAE4 and DCE4 from the control group (Figure S3C–D). This indicates the top genera, especially those before dacryocystorhinostomy, could potentially serve as signatures to distinguish the diseases from a healthy condition.

Furthermore, to distinguish DCAE and DCE separately from the control group before dacryocystorhinostomy, the top six important genera with the highest AUC of 0.900 and the top 29 important genera with the highest AUC of 0.913 were required (Figure S4A–B). After dacryocystorhinostomy, to separately distinguish DCAE4 and DCE4 from the control group, the top seven important genera with the highest AUC of 0.927 and the top 35 important genera with the highest AUC of 0.875 were needed (Figure S4C–D).

### Longitudinal comparison of ocular microbiota alterations during dacryocystorhinostomy treatment and recovery with and without antibiotic pretreatment

**Figure 4:**
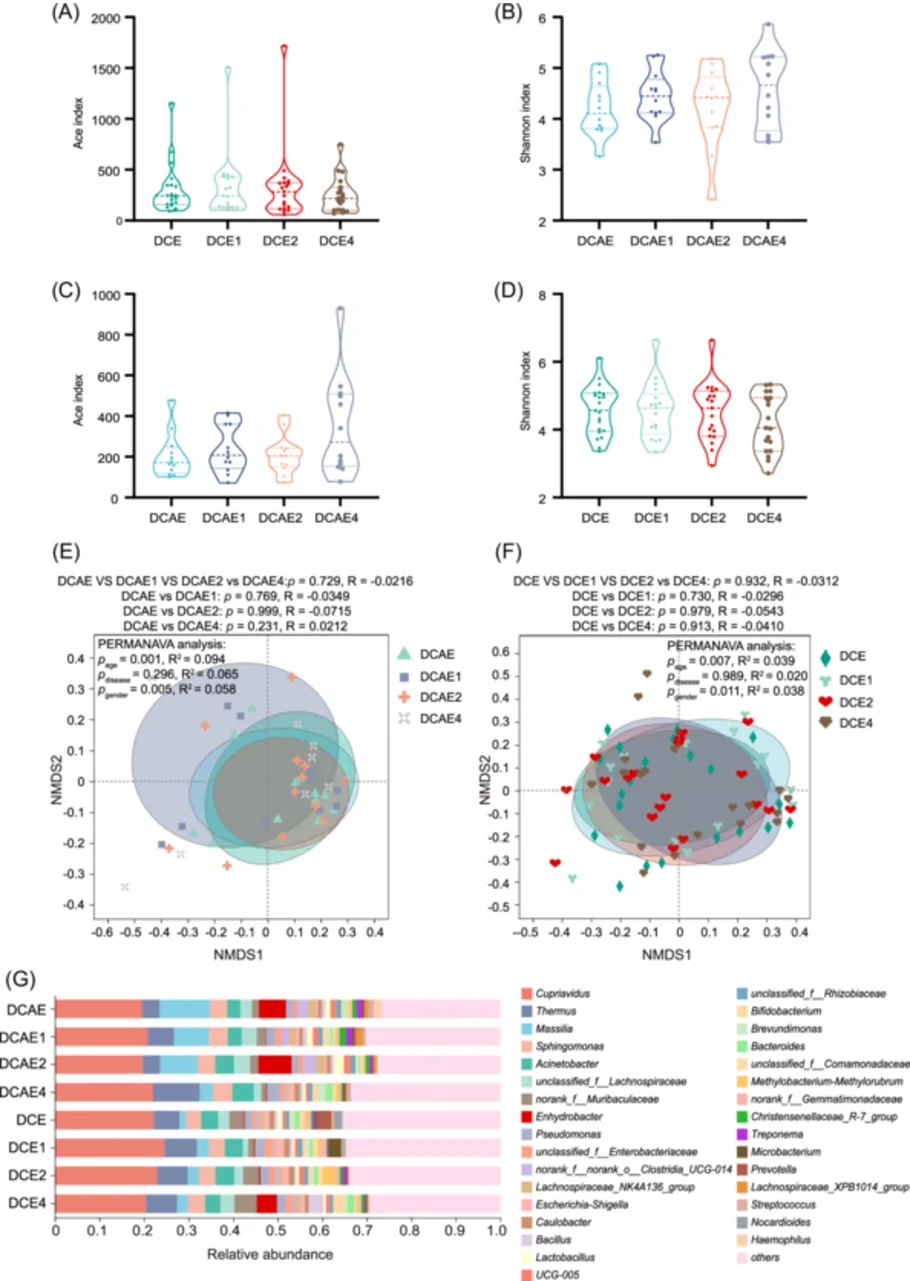
Longitudinal comparison of ocular microbiota of dacryocystitis (DC) patients with and without antibiotic pretreatment 1 day before (DCAE and DCE), and at the first, second, and fourth weeks after the dacryocystorhinostomy (DCAE1, DCAE2, DCAE4, and DCE1, DCE2, DCE4). (A) Comparison of ocular microbial alpha diversity with Ace index among DCAE, DCAE1, DCAE2, and DCAE4 groups. (B) Comparison of ocular microbial alpha diversity with Shannon index among DCAE, DCAE1, DCAE2, and DCAE4 groups. (C) Comparison of ocular microbial alpha diversity with Ace index among DCE, DCE1, DCE2, and DCE4 groups. (D) Comparison of ocular microbial alpha diversity with Shannon index among DCE, DCE1, DCE2, and DCE4 groups. (E) Comparison of ocular microbial beta diversity with the ANOSIM analysis based on Bray–Curtis distance matric among the DCAE, DCAE1, DCAE2, and DCAE4 groups. In addition, the PERMANOVA analysis considering age and gender effects was applied. (F) Comparison of ocular microbial beta diversity with the ANOSIM analysis based on Bray–Curtis distance matric among the DCE, DCE1, DCE2, and DCE4 groups. In addition, the PERMANOVA analysis considering the age and gender effects was applied. (G) The microbial genera composition of the DCAE, DCAE1, DCAE2, and DCAE4, as well as the DCE, DCE1, DCE2, and DCE4 groups. Only genera with relative abundance of more than 1% are listed.

**Figure 5:**
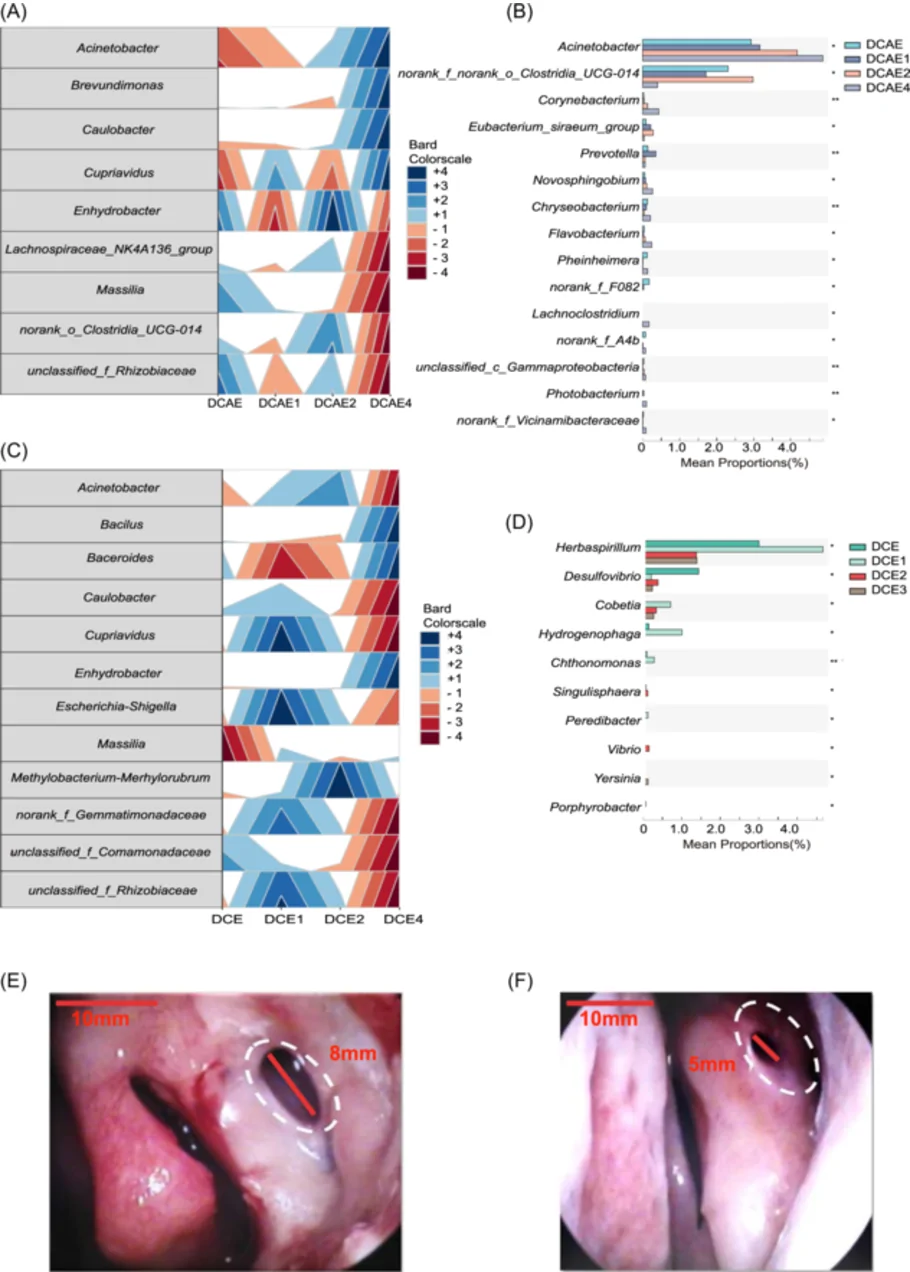
Fluctuation of ocular microbes in dacryocystitis (DC) patients with and without antibiotic pretreatment. (A) The alteration of key ocular bacteria during the time series of DCAE, DCAE1, DCAE2, and DCAE4 groups using BiomeHorizon horizon plot. Values are plotted as a relative abundance versus time area graph for each time point. The median relative abundance was centred to “zero” and referred to as the “origin” The darker blue bands indicate values incrementally above the origin and darker red bands below the origin. Only microbes with a per‐sample average abundance above 0.75% (thresh_abundance = 0.75) across all samples were chosen. (B) Significantly different genera when comparing the four‐time points of DC patients with antibiotic pretreatment. The Kruskal–Wallis test with Tukey–Kramer post hoc test was employed to test microbial alpha diversity differences. *FDR < 0.05, **FDR < 0.01, ***FDR < 0.001. (C) The alteration of key ocular bacteria during the time series of DCE, DCE1, DCE2, and DCE4 groups using BiomeHorizon horizon plot. Data is presented same as in (A). (D) Significantly different genera when comparing the four‐time points of DC patients without antibiotic pretreatment. The Kruskal–Wallis test with Tukey–Kramer post hoc test was employed to test microbial alpha diversity differences. *FDR < 0.05, **FDR < 0.01, ***FDR < 0.001. (E) Clinical image of nasal endoscopy of DC patients with antibiotic pretreatment at the fourth week after the dacryocystorhinostomy. (F) Clinical image of nasal endoscopy of DC patients without antibiotic pretreatment at the fourth week after the dacryocystorhinostomy. The opened anastomotic stoma is marked with a dashed circle.

To systemically analyse the effects of dacryocystorhinostomy on ocular microbiota changes and compare the differences between patients treated with and without antibiotics before surgery, we further analysed ocular microbiota changes at four follow‐up time points, pre‐ and post‐dacryocystorhinostomy. In both DC patients who did and did not undergo antibiotic pretreatment, no significant differences in microbiota diversity were observed among the different time points (Figure 4 and Figure S1G–J). The top bacterial genera in the antibiotic pretreatment group were *Cupriavidus*, *Massillia*, *Thermus*, *Sphingomonas*, *Acinetobacter*, and *Enhydrobacter* (Figure 4G). *Cupriavidus* slightly increased at Week 1, decreased at Week 2, and increased again by Week 4. Similar trends were observed for *Sphingomonas* and *Thermus. Conversely, the abundances of Enhydrobacter*, together with *the Lachnospiraceae* NK4A136 group and *Clostridia* UCG‐014, were the opposite; they were slightly lower at Week 1, increased at Week 2, and had a lower relative abundance at Week 4. The relative abundance of *Massillia* decreased, and that of *Acinetobacter* increased from Week 1 until the last time point (Figure 5A). In addition to these trends, other significant changes in the bacterial genera during the time points were also observed by Week 4 (Figure 5B). Different ocular microbes from each time point postdacryocystorhinostomy compared to the sample collected before the dacryocystorhinostomy with antibiotic pretreatment were also found (Figure S5A–C).

The top bacterial genera in the group not subjected to antibiotic pretreatment were also *Cupriavidus*, *Thermus, Acinetobacter, Sphingomonas*, and *Massillia* (Figure 4G). The abundances of both *Cupriavidus* and *Acinetobacter* increased at Weeks 1 and 2 and then decreased at Week 4. Quite a few bacteria, including *Escherichia‐Shigella* and *Gemmatimonadaceae*, exhibited similar trends (Figure 5C). *Thermus* and *Massillia* had higher relative abundances at Week 1 and were either stable or slightly decreased at Week 2 and increased at Week 4. *Sphingomonas* stably increased until the fourth week (Figures 4G and 5C). The abundance of other significantly changed genera, including *Herbaspirillum*, which increased beginning at Week 1 but decreased to a lower level than that before dacryocystorhinostomy beginning at Week 2, was low. *Desulfovibrio* had the highest abundance before dacryocystorhinostomy and decreased beginning at Week 1 (Figure 5D). Differences in microbial composition at each follow‐up time after dacryocystorhinostomy compared to that in the sample collected before antibiotic pretreatment were also analysed (Figure S5D–F).

In summary, the changes in bacterial abundance in the longitudinal samples were different between the groups with and without antibiotic pretreatment. The alteration tendencies of *Acinetobacter*, *Massilia*, and *Cupriavidus* were opposite in the DC patients with or without antibiotic pretreatment, while the alteration tendencies of *Pseudomonas*, *Sphingomonas*, and *Thermus* were similar (Figure 5A,C).

### The clinical recovery of DC patients, with and without antibiotic pretreatment, after dacryocystorhinostomy was similar

At the fourth week after dacryocystorhinostomy, the lacrimal sac was directly anastomosed with the nasal tract in all patients without obstruction (Table S2). In the first week, 10 out of 19 patients in the DC group and five out of 12 patients in the DCA group experienced symptoms of lacrimation (*p* = 0.5518). In the second week, this number decreased to two for each of the DC and DCA groups (*p* = 0.6194) (Table S2). By the fourth week, clinical examination revealed no symptoms of lacrimation or ocular discharge from DC patients either with (Figure 5E and Table S2) or without antibiotic pretreatment (Figure 5F and Table S2). Hence, antibiotic pretreatment will not affect the recovery of DC patients after endoscopic dacryocystorhinostomy.

### Two ocular microbiota types were identified in the ocular microbiota

**Figure 6:**
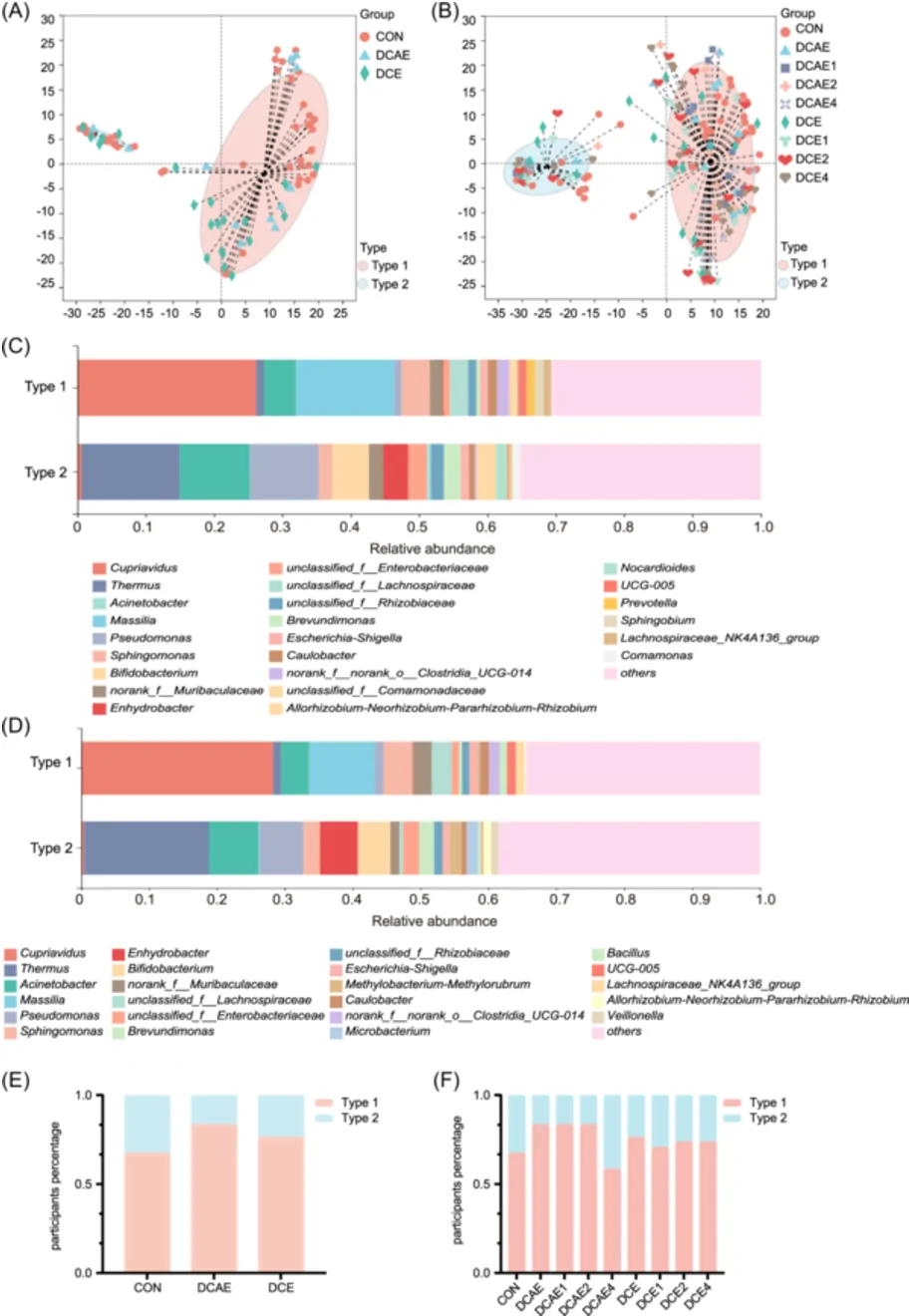
Ocular microbial types and their distribution in healthy participants and dacryocystitis (DC) patients. (A) Using partitioning around medoids (PAM) clusters based on the Jensen‐Shannon distance (JSD) and Calinski–Harabasz (CH) indices, two different ocular microbial types were identified with the genera from CON, DCAE, and DCE groups. (B) Two different ocular microbial types were identified based on the genera from CON participants and all DC patients. (C) The microbial genera composition of the two different ocular microbial types based on CON, DCAE, and DCE groups. Only genera with relative abundance of more than 1% are listed. (D) The microbial genera composition of the two different ocular microbial types based on CON participants and all DC patients. Only genera with relative abundance of more than 1% are listed. (E) The prevalence of the two ocular microbial types in CON, DCAE, and DCE groups. (F) The prevalence of the two ocular microbial types among CON and all DC patients.

The ocular microbiota tended to form two or three clusters according to the beta diversity analysis. To evaluate this phenomenon, ocular microbiota types were analysed and identified. Interestingly, two ocular microbiota types were identified, regardless how we took our groups (Figure 6A,B and Figure S6A). Type 1 contained a high abundance of *Cupriavidus* and *Massilia*, and type 2 contained more *Thermus* and *Acinetobacter* (Figure 6C,D and Figure S6B). Although we observed a higher prevalence of type 1 among DC patients than healthy participants, there was no significant distribution difference among the groups (Figure 6E,F). Notably, the distribution of ocular microbiota types was different among DC patients compared to other ocular diseases, with the latter containing a higher prevalence of type 2 ocular microbiota (Figure S6C).

### The nasal microbiota was comparable to the ocular microbiota and affected by antibiotic pretreatment in DC patients before dacryocystorhinostomy

In addition to ocular microbiota, we also collected nasal swab samples from all DC patients before and at the fourth week after dacryocystorhinostomy to study how the nasal microbiota is related to the ocular microbiota and whether it is influenced by antibiotic retreatment. A comparison of the ocular and nasal microbiota indicated similar microbiota diversity (Figure S7A–C) and composition (Figure S7D). Moreover, significantly decreased *Thermus* and *Ruminococcaceae* UCG‐005 and significantly increased *Streptococcus* were identified when compared to the nasal microbiota with ocular microbiota (Figure S7E).

**Figure 7:**
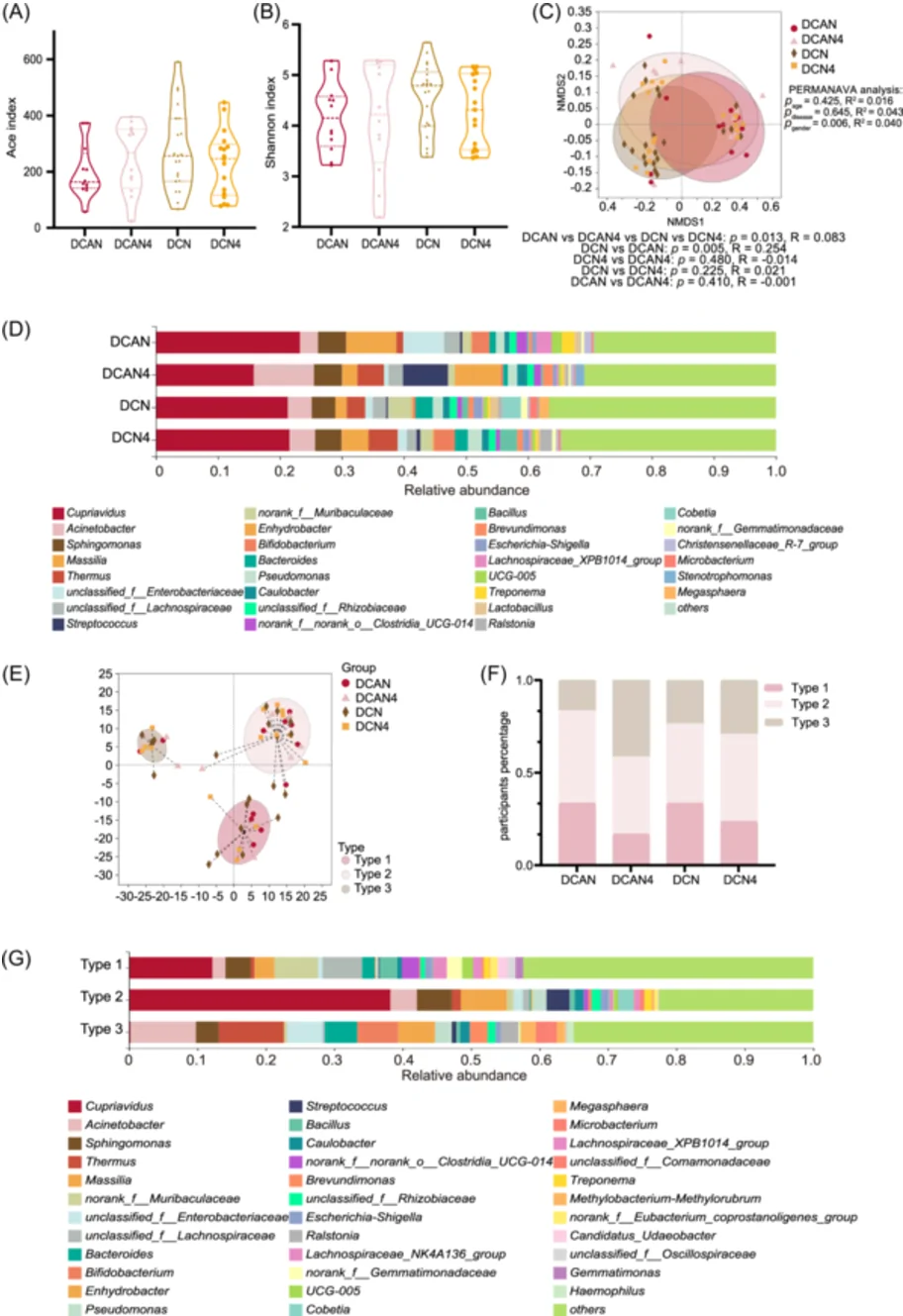
Nasal microbiota in dacryocystitis (DC) patients and the distribution of the nasal microbial types. (A) Comparison of nasal microbial alpha diversity with Ace index among DC patients with (DCAN) and without (DCN) antibiotic pretreatment before and at the fourth week after the dacryocystorhinostomy (DCAN4 and DCN4). (B) Comparison of nasal microbial alpha diversity with Shannon index among DCAN, DCAN4, DCN, and DCN4 groups. The Kruskal–Wallis test with Tukey–Kramer post hoc test was employed to test microbial alpha diversity differences. (C) Comparison of nasal microbial beta diversity with the ANOSIM analysis based on Bray–Curtis distance matric among the DCAN, DCAN4, DCN, and DCN4 groups. In addition, the PERMANOVA analysis considering age and gender effects was applied. (D) The microbial genera composition of the DCAN, DCAN4, DCN, and DCN4 groups. Only genera with relative abundance of more than 1% are listed. (E) Three different nasal microbial types were identified based on the genera from DCAN, DCAN4, DCN, and DCN4 groups. (F) The microbial genera composition of the three different nasal microbial types based on DCAN, DCAN4, DCN, and DCN4 groups. Only genera with relative abundance of more than 1% are listed. (G) The prevalence of the three nasal microbial types in DCAN, DCAN4, DCN, and DCN4 groups.

When we investigated nasal microbiota with and without antibiotic pretreatment before and 4 weeks postdacryocystorhinostomy, no significant alteration of nasal microbiota was identified (Figure 7A,B and Figure S1K–L). The comparison of beta diversity revealed a significant dissimilarity (*p*_ANOSIM_ = 0.013) among the four groups, which was mainly attributed to the differences between the groups with and without antibiotic pretreatment before dacryocystorhinostomy (Figure 7C). However, according to the PERMANOVA of the comparison of microbiota beta diversity, no significant difference in the microbiota was observed after excluding the potential interferences from age and gender (Figure 7C). The top five main nasal microbiota genera were same as the top identified genera in ocular microbiota (Figure 7D).

Based on the similarity of the nasal genera, a total of three nasal microbiota types were identified (Figure 7E). Type 1 included *Cupriavidus*, *Muribaculaceae*, and *Lachnospiraceae*; type 2 included *Cupriavidus* and *Massillia*; and type 3 included *Acinetobacter* and *Thermus* (Figure 7G). The three nasal microbiota types were distributed similarly among all the groups (Figure 7F). Furthermore, a comparison of each paired nasal microbiota group performed with different nasal microbes were found (Figure S8).

## 3 DISCUSSION

In this study, we investigated the relationship between ocular microbiota composition and various ocular diseases, including DC, DCC, SLC, and MALT patients, with a focus on longitudinal ocular microbiota changes between patients with and without antibiotic pretreatment. We demonstrated the altered ocular microbiota β‐diversity between DC or MALT patients and healthy individuals. Further analysis showed a higher microbiota diversity in the MALT patients and the DC patients without antibiotic pretreatment than the healthy control participants, which were consistent with previous results that the ocular microbiota diversity is higher in patients with ocular diseases [13, 39, 40]. Notably, although the clinical recovery rates were comparable, our results suggest that, after the dacryocystorhinostomy, the richness and diversity of the ocular microbiota in DC patients with antibiotics pretreatment significantly increased at the 4th week after surgery, which suggests that the recovery of their ocular microbiota to healthy condition may be more disturbed than that in patients not receiving antibiotic pretreatment. Our findings are in line with those of several previous studies indicating that antibiotic treatment negatively affects the surface of the eye microbiota, potentially contributing to the opportunistic invasion of pathogenic species and eye diseases and promoting the development of drug resistance [8, 15, 35].

The ocular microbiota has been very rarely studied, especially in longitudinal samples. Our study provides comprehensive ocular microbiota data, which are of significant value for future studies. *Acinetobacter*, *Cupriavidus*, and *Caulobacter*, whose abundances are often significantly reduced in DC, MALT, and DCC patients, were suggested to be important resident ocular bacteria in previous studies [11, 41, 42, 43, 44, 45, 46]. Furthermore, *Streptococcus* [47, 48], *Pelomonas* [49, 50], and *Bacillus* [48, 51], which were significantly enriched in ocular diseases in our study, were shown to be significantly enriched in other ocular diseases and to induce intraocular inflammation. Taken together, our results indicate that these significantly increased genera may be involved in the underlying mechanisms that are beneficial for the proliferation of opportunistic pathogens.

The identification of the key differential genera in longitudinal samples could help us to study the potential effects of ocular microbiota on the disease recovery process. For example, similar increases in *Pseudomonas*, *Sphingomonas*, and *Thermus* [11, 52], which are widely suggested to be the most abundant resident ocular bacteria in healthy humans, indicated that the recovery of ocular microbiota is accompanied by the recovery of DC patients after dacryocystorhinostomy. The significant increase in *Acinetobacter* and *Cupriavidus* in the DCAE group and *Massilia* in the DCE group, which are also abundant resident ocular bacteria among healthy individuals suggested a potential differential ocular microbiota recovery pathways [53].

Two ocular microbiota types were proposed for the first time in our study. These two types are commonly found in both healthy individuals and DC patients, but with higher percentage of type 2 among healthy individuals. The two ocular microbiota types do not frequently change since there were only two patients out of all the DC patients belonging to type 2 before the dacryocystorhinostomy, who were changed to type 1 at the fourth week after the dacryocystorhinostomy. However, whether the type 1 ocular microbiota contains certain microbes or networks involved in DC development needs further investigation in larger cohorts.

The development of sequencing techniques and modelling algorithms has facilitated the use of microbiota to predict the risk of disease, which is one clinical application of microbiome research [54]. However, studies on the prediction of ocular disease onset based on the ocular microbiota are still limited. In this study, the identified genera signatures' ability to distinguish DC patients from healthy volunteers before and after dacryocystorhinostomy suggests that the ocular microbiota may assist in predicting ocular diseases and monitoring patients' recovery. By comparing the number of feature genera distinguishing DCAE from the control group and distinguishing DCE from the control group (29 vs. 6 before dacryocystorhinostomy and 35 vs. 7 after dacryocystorhinostomy), these results indicate that the ocular microbiota is more similar between DC patients without antibiotic pretreatment and healthy controls. This finding supports our major clinical suggestion that antibiotic pretreatment may not be necessary before dacryocystorhinostomy for DC patients, especially for non‐severe cases.

The nasal microbiota related to antibiotic pretreatment and dacryocystorhinostomy were also investigated. We found that the nasal microbiota diversity and composition of DC patients with or without the antibiotic pretreatment were similar. Furthermore, we demonstrated that the nasal microbiota is very comparable to the ocular microbiota. This finding suggested that ocular and nasal samples could be replaced by each other when needed. The nasal microbiota may serve as the bacterial source after antibiotic pretreatment or dacryocystorhinostomy and assist in reestablishment of the ocular microbiota [55].

Overall, considering the longitudinal analysis of ocular and nasal microbiota obtained during DC dacryocystorhinostomy treatment and during recovery with or without antibiotic pretreatment, we found that there was no significant difference in recovering from DC between the two groups. Notably, both the α diversity and random forest analyses indicated that antibiotic pretreatment may lead to greater differences in the ocular microbiota between postsurgical patients and healthy volunteers, suggesting that antibiotic pretreatment may not be conducive to recovery of the ocular microbiota after surgery. Considering the overuse of antibiotics may lead to dysbiosis of the local microbiota and increase the likelihood of antibiotic resistance transmission [37, 38], we suggest to re‐evaluate the antibiotic use for each DC patients before dacryocystorhinostomy treatment.

This study has several limitations. First, while our cohort is one of the most longitudinal cohorts for ocular diseases, the total number of patients was still limited, comprising samples from only one hospital, and including urban residents as well as those from surrounding rural areas. This limitation might affect the statistical significance and application of our data, which must be considered when interpreting our results and should be validated in a larger cohort. Second, although we attempted to reduce potential contamination during sampling and sequencing by using nuclease‐free water as the negative control, future ocular microbiota research should consider including more negative controls, such as hospital air swabs and periocular skin samples. Third, we utilized 16S rRNA gene sequencing, which has limited taxonomic classification at the species level and does not provide information on microbial functions. Fourth, we employed the random forest algorithm to screen for potential microbial signatures for identifying patients with DC. External validation from similar cohorts will strengthen our findings. Finally, we only followed the patients for 4 weeks after dacryocystorhinostomy, which limited our ability to study potential recurrence or infection after this period. However, given the rarity of studies on the ocular microbiota, our study still represents an important longitudinal cohort study that provides comprehensive data and information following the recovery of DC patients.

## 4 CONCLUSION

Overall, this study identified two common ocular microbiota types (with most common genera as *Cupriavidus* or *Thermus*) among ocular disease patients. The recovery of DC patients after dacryocystorhinostomy with and without antibiotic pretreatment was similar; however, the ocular microbiota with the antibiotics pretreatment is significantly changed at the fourth week postdacryocystorhinostomy. Antibiotic‐resistant bacteria rapidly spread worldwide and threaten millions of lives [56, 57]; reducing unnecessary antibiotic usage is essential and highly valuable. Our study supports the discontinuation of antibiotics for DC patients before dacryocystorhinostomy. This contributes to the critical “One Health” issue and reduces antibiotic abuse and the emergence of antibiotic‐resistant microorganisms.

## 5 METHODS

### Study design, population, diagnosis, and sample collection

This study sample comprised 47 patients who visited Tianjin Medical University Eye Hospital, China due to ocular discomfort from November 2018 to August 2019 (Figure 1A). Among these 47 patients, 33, 5, 4, and 5 patients were diagnosed with chronic DC, chronic DCC, SLC, and ocular MALT involving the conjunctiva, respectively, according to clinical signs and histopathology [58, 59, 60, 61, 62, 63, 64]. In addition, conjunctival sac swab samples from 46 healthy volunteers without eye disease or other detected diseases were also collected during the same period; these volunteers composed the control group (CON). The 33 DC patients were further divided into two groups according to their previous antibiotic usage. Twelve DC patients who were pretreated with levofloxacin eye drops following the doctor's prescription were grouped into the chronic DC antibiotic pretreatment (DCA) group, in which conjunctival sac swab samples were labelled DCAE and nasal samples were labelled DCAN. In addition, another 21 chronic DC patients without antibiotic pretreated were grouped into the DC DCE group for conjunctival sac swab samples and the DCN group for nasal samples (Figure 1A). All patients with DC, DCC, SLC, or MALT, as well as the healthy volunteers, did not receive any medicines during the study period, except for the use of levofloxacin eye drops in the DCA group.

All 33 DC patients were treated with dacryocystorhinostomy and were followed‐up for 4 weeks. Briefly, surgery was conducted under local anaesthesia combined with monitored anesthesia care. The upper limit is located in front of the middle turbinate's attachment point, while the lower limit is the inferior margin of the middle turbinate. A “[”‐shaped nasal mucosal flap was created using a crescent blade. The flap was separated along the periosteal surface, turned backward, and positioned in the middle nasal canal, revealing the lacrimal bone and the maxillary frontal process. Bone biting forceps were used to grip the bone on the inner posterior wall of the lacrimal sac fossa, creating a bone window approximately 15 × 10 mm² in size, which exposed the inner wall of the lacrimal sac. A probe was inserted into the lower lacrimal punctum to explore the incision in the lacrimal sac. The lacrimal sac was incised from the upper edge of the bone window down to the lower end of the lacrimal sac. The nasal mucosa was repositioned, overlapped with the lacrimal sac mucosa, and excess nasal mucosa and lacrimal sac mucosa were trimmed off. This allowed the free edges of the mucosa to be joined smoothly and attached. A hemostatic sponge was placed over the anastomosis of the nasal mucosal flap and the lacrimal sac mucosal flap, filling the middle nasal meatus.

Conjunctival sac swab samples were collected 1 day before dacryocystorhinostomy, and the first, second, and fourth weeks after the dacryocystorhinostomy. These samples were named DCE, DCE1, DCE2, and DCE4 for the group without antibiotic pretreatment, and DCAE, DCAE1, DCAE2, and DCAE4 for the group with antibiotic pretreatment. In addition, nasal swab samples were collected from all DC patients 1 day before and at the fourth‐week visit, and were named DCN and DCN4 for patients without antibiotic pretreatment, DCNA and DCNA4 for the ones with antibiotic pretreatment. Further, the medical records for all the DC patients were collected, including the tear and discharge information, as well as the tear duct blockage status at the fourth week after the dacryocystorhinostomy. At the time of diagnosis, swab samples were cultured on GC agar plates (BIO‐KONT) to test for common eye infection pathogens, including *Streptococcus pyogenes*, *Streptococcus pneumoniae*, *Staphylococcus aureus*, and *Escherichia coli*. Further, at the fourth week after the dacryocystorhinostomy, the clinical images of nasal endoscopy of all DC patients were taken under nasal endoscope (N‐90×0568‐G, KARL STORZ‐ENDOSKOPE) to detect the recovery situation after dacryocystorhinostomy.

Participant characteristics of the 46 healthy volunteers and 47 patients are listed in Table S1. No participant reported any ocular disease, such as blepharitis, hordeolum, entropion, ectropion, or corneal disease, other than the diagnosed DC, DCC, SLC, or MALT within 3 months. The exclusion criteria included participants who: (1) had taken antibiotics other than the prescribed levofloxacin eye drops within the past month, (2) had undergone glucocorticoid therapy within the previous 3 months, (3) were suffering from severe skin diseases such as acne and rosacea, (4) had diabetes, autoimmune diseases, renal insufficiency requiring dialysis, or other systemic diseases, (5) had nasal conditions such as rhinopolyps, atrophic rhinitis, or a recent history of nasal surgery, (6) had a medical or allergy history that could affect the test results, and (7) had participated in other clinical studies within the last 3 months.

Before ocular microbiota sampling, one drop of proparacaine eye drop was applied as local anaesthesia. A dry, sterile swab (ZYMO) was used for wiping the front, central, and lateral fornix of the lower eyelid conjunctival sac three to four times to maximize bacterial load on the swab and avoid contamination by avoiding contact with eyelashes. For the nasal microbiota sample collection, a nose trimmer and entoiodine clear nasal vestibule were used before surgery. Nasal microbiota sampling was performed by inserting the swab into the middle meatus, gently wiping the origin of the middle turbinate near the nasal mound thrice, avoiding contact with the anterior nasal mucosa and nasal vestibule, to maximize bacterial load on the swab and avoid contamination. After sampling, the swabs were quickly put in the collection tube with DNA/RNA shield (ZYMO) and then stored at −80°C until laboratory preparation [6].

### DNA extraction and 16S rRNA gene sequencing

The total DNA from the collected swab samples was extracted using the Mag‐Bind Soil DNA kit (Omega Bio‐tek). Nuclease‐free water was used as control. DNA quality was assessed by 0.8% agarose gel electrophoresis. The final DNA concentration and purification were determined by fluorometry using a Qubit 2.0 fluorometer (Life Technologies). The total DNA was eluted in 50 μL of DNase‐free water and stored at −20°C until further library preparation and 16S rRNA gene sequencing.

The V3–V4 regions of the 16S rRNA genes were amplified with Illumina sequencing index‐binding primer pairs 338 F (5′‐ACTCCTACGGGAGGCAGCA‐3′) and 806 R (5′‐GGACTACHVGGGTWTCTAAT‐3′) using the following polymerase chain reaction (PCR) conditions: 15 s at 98°C, 30 s at 55°C, and 30 s at 72°C for 27 cycles. PCRs were performed with 5 μL 5× reaction buffer, 5 μL 5× GC buffer, 2 μL 2.5 mM deoxynucleoside triphosphates (dNTPs), 1 μL of each primer (10 μM), 0.25 μL Q5 DNA Polymerase, 40 ng of DNA template, and extra ddH_2_O in a 25 μL system. Nuclease‐free water and extraction control was used as negative controls. Agarose gel electrophoresis was performed to verify the size of amplicons. Finished libraries were quantified using Quant‐iT fluorometric assay (Thermo Fischer Scientific) and Agilent Bioanalyzer (Agilent) [65]. Thereafter, paired‐end sequences (2 × 300 bp) of the prepared sample libraries were generated on an Illumina MiSeq sequencing platform (Illumina) with MiSeq Reagent Kit v3 (Illumina).

### Bioinformatics analysis

#### Taxonomic assignment

Raw reads were demultiplexed and processed (e.g., quality filtering and trimming) using tools available in QIIME 2 (version 2020.8) [66]. Clean reads were then denoised and clustered with DADA2 [67]. To minimize the effects of sequencing depth on alpha and beta diversity measure, the number of sequences from each sample was rarefied to the lowest read numbers of detected samples. Taxonomic assignment of amplicon sequence variants (ASVs) (Figure S1) was performed using the naive‐Bayes consensus taxonomy classifier [68] implemented in QIIME2 and the SILVA 138 database. Microbes are labelled by their most fine‐grained level of taxonomic identification.

#### Alpha and beta diversity

The following analysis on alpha and beta diversity was performed on the filtered data using “q2‐diversity” commands in QIIME2 [68, 69, 70]. Notably, to minimize the effects of sequencing depth on alpha and beta diversity measure, the number of sequences from each sample was rarefied to the lowest reads number of detected samples. In detail, ASV richness estimates (Ace) and diversity indices (Shannon) were carried out for microbiota alpha diversity. Beta diversity was carried out via nonmetric multidimensional scaling (NMDS) analysis based on Bray–Curtis dissimilarity. In addition, PERMANOVA with different age, gender, and disease types were performed to compare the contribution of each effector and their covariate to the microbiota difference. Furthermore, Partitioning Around Medoids (PAM) clustering was performed based on the Jensen‐Shannon divergence (JSD). The best clustering K number was calculated using the Calinski‐Harabasz (CH) index [71]. The microbiota types were analysed using between‐class analysis (BCA).

#### Random forest analysis

The randomForest package in R was used for random forest analysis [72], with the identified ocular and nasal bacteria being used as the inputs of the random forest model to classify DCAE and DCE with control group, as well as DCAE4 and DCE4 with control group. To further minimize the potential over‐fitting in the model, a machine learning method with a 10‐fold cross‐validation approach (“trainControl” package in R) was applied for the compassion among three groups [72]. The method splits all samples into the train model subsets and the test model subset, which is totally different from the test model subset. The 10‐fold cross‐validation was completed until accuracy was determined for each permutation and combination of samples in the subsets, and then an overall accuracy was estimated [72]. Further, Receiver operating characteristic (ROC) analysis was performed to measure the quality of the classification models between two groups by the R software package, pROC (v1.16.2). ROC curve results were plotted by the true positive rate against the false positive rate, and the area under curve (AUC) was used to designate the ROC effect. The AUC of the optimized model was calculated using “roc.curve” package in R.

#### Microbiome time series

Based on the identified ASV tables, BiomeHorizon (https://github.com/blekhmanlab/biomehorizon/) was used to identify the microbiota changes over time with default parameter settings [73]. BiomeHorizon is used to detect the key microbes related to patients' recovery process and reveal links between the time factors and microbial dynamics.

### Statistics

The Chi‐square test was performed to compare the cured effects of antibiotic pretreatment before the endoscopic dacryocystorhinostomy. The Mann–Whitney *U* test with multiple comparisons adjusted by the Benjamini–Hochberg FDR was performed to compare the microbial alpha diversity of two groups. The Kruskal–Wallis test with the Tukey–Kramer post hoc test adjusted by the Benjamini–Hochberg FDR was employed to test microbial alpha diversity differences in more than two groups [74]. ANOSIM analysis based on Bray–Curtis distance matrices was used to identify the beta diversity between the groups. Adonis function from the R package “vegan” was used for the PERMANOVA analysis [75, 76].

## AUTHOR CONTRIBUTIONS

**Shengru Wu**: Investigation; conceptualization; methodology; software; data curation; supervision; validation; writing—original draft; writing—review and editing; visualization. **Limin Zhu**: Methodology; conceptualization. **Tingting Wang**: Conceptualization; methodology; supervision. **Chenguang Zhang**: Methodology. **Jiaqi Lin**: Methodology; supervision. **Yanjin He**: Resources. **Junhu Yao**: Methodology; supervision. **Tingting Lin**: Conceptualization; methodology; supervision; data curation; resources; project administration; writing—review and editing; visualization; funding acquisition. **Juan Du**: Writing—original draft; conceptualization; methodology; funding acquisition; project administration; supervision.

## CONFLICT OF INTEREST STATEMENT

The authors declare no conflict of interest.

## ETHICS STATEMENT

The ethics application (No. 2018KY‐09) was approved by the Regional Ethical Board at the Tianjin Branch of the National Clinical Research Center for Ocular Disease, Tianjin, China, and all participants provided written informed consent to participate in this study.

## Supporting information


**Figure S1:** Identification and comparison of ocular or nasal microbiota ASVs types and total numbers of ASVs in different ocular diseases.
**Figure S2:** Comparison of ocular microbiota of the control group with ocular microbiota from DC patients with and without antibiotic pre‐treatment (named as DCAE‐ALL and DCE‐ALL groups), by involving all conjunctival sac swab samples that collected one day before dacryocystorhinostomy, and the first, second, and fourth weeks after the dacryocystorhinostomy.
**Figure S3:** The identification of potential biomarker to distinguish chronic dacryocystitis (DC) patients with (DCAE) and without (DCE) the antibiotic pre‐treatment before the dacryocystorhinostomy, and at the fourth week after the dacryocystorhinostomy (DCAE4 and DCE4).
**Figure S4:** The identification of potential biomarker to separately distinguish patients' ocular samples of DCAE DCE, DCAE4 and DCE4 groups from the healthy volunteers' ocular samples of the CON group.
**Figure S5:** Significantly different genera when comparing each pair of the four time points with and without antibiotic pre‐treatment in DC patients.
**Figure S6:** Two ocular microbial types and their distribution in different ocular diseases patients.
**Figure S7:** Comparison of ocular and nasal microbiota of DC patients.
**Figure S8:** Significantly different genera when comparing nasal microbiota from DC patients with (DCAN) and without (DCN) antibiotic pre‐treatment, both before and at the fourth week after the dacryocystorhinostomy (DCAN4 and DCN4).

**Table S1:** Clinical characteristics of the healthy participants and patients with different ocular diseases.
**Table S2:** The clinical recovery data of all chronic dacryocystitis patients with or without antibiotic pre‐treatment.

## Data Availability

All the data generated or analysed for this study are included in this paper. The data and scripts used are saved in GitHub https://github.com/shengru-wu/Wu2024imetaomics. The sequencing reads are available in the Sequence Read Archive (SRA) of NCBI under accession project number PRJNA874534 (https://www.ncbi.nlm.nih.gov/bioproject/874534). Supplementary materials (figures, tables, scripts, graphical abstract, slides, videos, Chinese translated version and update materials) may be found in the online DOI or iMeta Science http://www.imeta.science/imetaomics/.
