# Supplementary material for: Ocular microbiota types and longitudinal microbiota alterations in patients with chronic dacryocystitis with and without antibiotic pretreatment
Source: IMetaOmics. 2024 Jul 9;1(1):e17. doi: 10.1002/imo2.17 (PMC12806523; doi:10.1002/imo2.17)
Supplement: Supplementary file 1 — Figure S1: Identification and comparison of ocular or nasal microbiota ASVs types and total numbers of ASVs in different ocular diseases. Figure S2: Comparison of ocular microbiota of the control group with ocular microbiota from DC patients with and without antibiotic pre‐treatment (named as DCAE‐ALL and DCE‐ALL groups), by involving all conjunctival sac swab samples that collected one day before dacryocystorhinostomy, and the first, second, and fourth weeks after the dacryocystorhinostomy. Figure S3: The identification of potential biomarker to distinguish chronic dacryocystitis (DC) patients with (DCAE) and without (DCE) the antibiotic pre‐treatment before the dacryocystorhinostomy, and at the fourth week after the dacryocystorhinostomy (DCAE4 and DCE4). Figure S4: The identification of potential biomarker to separately distinguish patients' ocular samples of DCAE DCE, DCAE4 and DCE4 groups from the healthy volunteers' ocular samples of the CON group. Figure S5: Significantly different genera when comparing each pair of the four time points with and without antibiotic pre‐treatment in DC patients. Figure S6: Two ocular microbial types and their distribution in different ocular diseases patients. Figure S7: Comparison of ocular and nasal microbiota of DC patients. Figure S8: Significantly different genera when comparing nasal microbiota from DC patients with (DCAN) and without (DCN) antibiotic pre‐treatment, both before and at the fourth week after the dacryocystorhinostomy (DCAN4 and DCN4). [file IMO2-1-e17-s002.docx]

**Supporting information to**

**Ocular microbiota types and longitudinal microbiota alterations in patients with chronic dacryocystitis with and without antibiotic pre-treatment**

**Running title**: Ocular microbiota and chronic dacryocystitis

Shengru Wu^1,3#^, Limin Zhu^2#^, Tingting Wang^2,4#^, Chengguang Zhang^1^, Jiaqi Lin^2^, Yanjin He^2^, Junhu Yao^1^, Tingting Lin^2^*, Juan Du^3^

^1^College of Animal Science and Technology, Northwest A&F University, Yangling 712100, China

^2^Tianjin Key Laboratory of Retinal Functions and Diseases, Tianjin Branch of National Clinical Research Center for Ocular Disease, Eye Institute and School of Optometry, Tianjin Medical University Eye Hospital, Tianjin 300384, China

^3^Department of Microbiology, Tumor and Cell Biology, Karolinska Institutet, Solna 17165, Sweden

^4^Department of Ophthalmology of the First Hospital of Xi’an, Shanxi Ophthalmological Institute, Xi‘an 710002, China.

^#^These authors contributed equally: Shengru Wu, Limin Zhu, Tingting Wang

*Corresponding author: [ltt6123@126.com](mailto:ltt6123@126.com) (Tingting Lin)

**Figure S1** **Identification and comparison of ocular or nasal microbiota ASVs types and total numbers of ASVs in different ocular diseases.** (A and B) Comparison of ocular microbiota ASVs types and total numbers of ASVs among DC-ALL, MALT, SLC, and DCC patients and healthy control group (CON). (C and D) Comparison of ocular microbiota ASVs types and total numbers of ASVs among CON, DCAE, and DCE groups. (E and F) Comparison of ocular microbiota ASVs types and total numbers of ASVs among CON, DCAE4, and DCE4 groups. (G and H) Comparison of ocular microbiota ASVs types and total numbers of ASVs among DCAE, DCAE1, DCAE2, and DCAE4 groups. (I-J) Comparison of ocular microbiota ASVs types and total numbers of ASVs among DCE, DCE1, DCE2, and DCE4 groups. (K and L) Comparison of nasal microbiota ASVs types and total numbers of ASVs among DCAN, DCAN4, DCN, and DCN4 groups. The Kruskal–Wallis test with Tukey–Kramer post hoc test was employed to test microbial alpha diversity differences. * FDR < 0.05.

**Figure S2** **Comparison of ocular microbiota of the control group with ocular microbiota from DC patients with and without antibiotic pre-treatment (named as DCAE-ALL and DCE-ALL groups), by involving all conjunctival sac swab samples that collected one day before dacryocystorhinostomy, and the first, second, and fourth weeks after the dacryocystorhinostomy.** (A) Comparison of ocular microbial alpha diversity with Ace index among CON, DCAE-ALL, and DCE-ALL groups. (B) Comparison of ocular microbial alpha diversity with Shannon index among CON, DCAE-ALL, and DCE-ALL groups. The Kruskal–Wallis test with Tukey-Kramer post hoc test was employed to test microbial alpha diversity differences. * FDR < 0.05. (C) Comparison of ocular microbial beta diversity with the ANOSIM analysis based on Bray–Curtis distance matric among the CON, DCAE, and DCE groups. In addition, P values that were calculated by repeated measures aware PERMANOVA adjusting for the age and gender effects.

**Figure S3 The identification of potential biomarker to distinguish chronic dacryocystitis (DC) patients with (DCAE) and without (DCE) the antibiotic pre-treatment before the dacryocystorhinostomy, and at the fourth week after the dacryocystorhinostomy (DCAE4 and DCE4).** (A) Using Random Forest analysis and the 10-fold cross-validation machine learning approach, the presented model evaluated the top important features to distinguish the DC patients from the CON before the dacryocystorhinostomy. (B) The top important genera to distinguish the CON, DCAE, and DCE groups. (C) The top important features to distinguish the DC patients from the CON at the fourth week after the dacryocystorhinostomy. (D) The top important genera to distinguish the CON, DCAE4, and DCE4 groups. The X-axis represents the number of variables (genera) of the importance ranking TOPn, and the Y-axis represents the average prediction error rate using the corresponding number of variables (genera). The dots presented the lowest error rate.


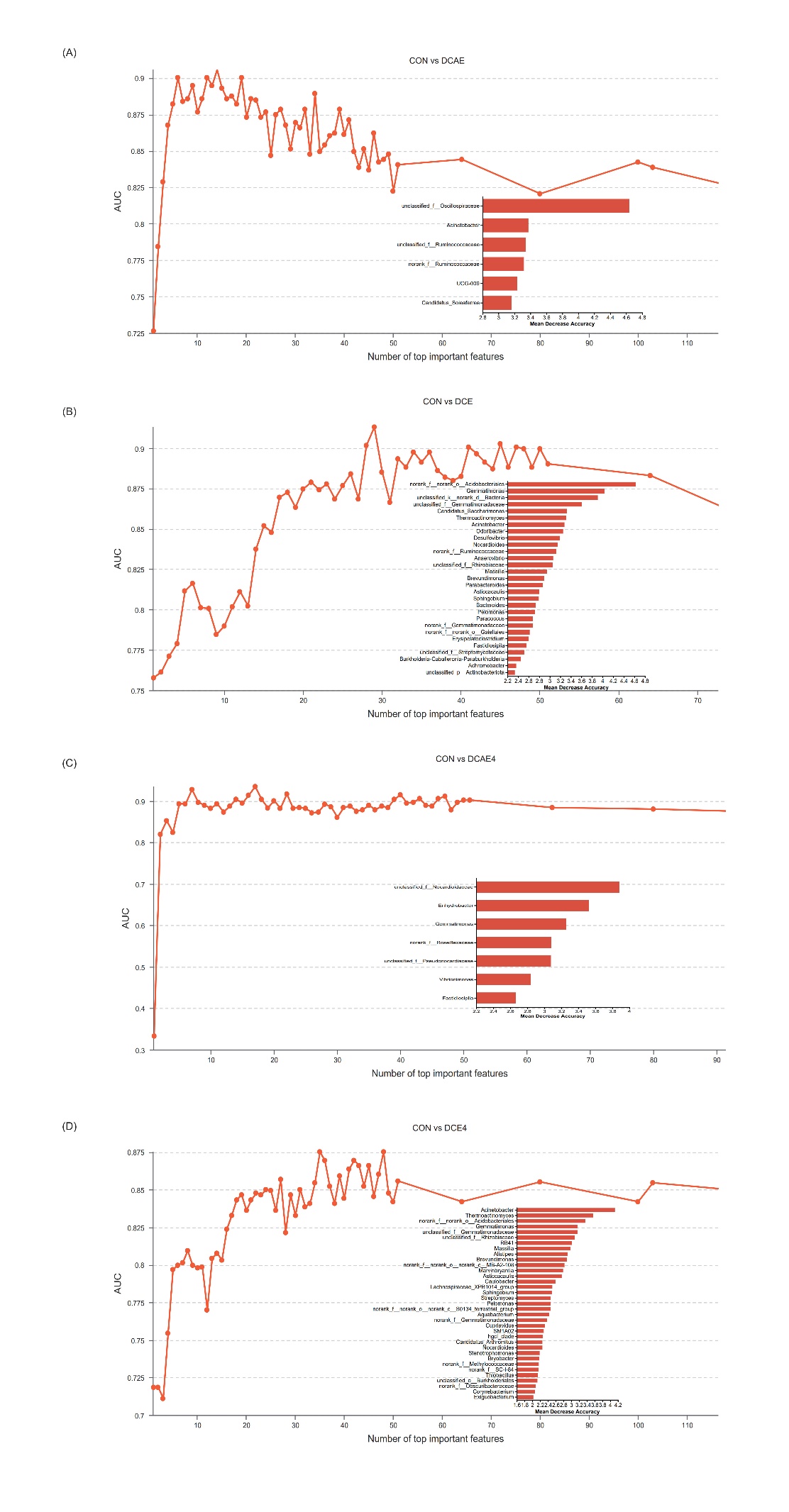


**Figure S4** **The identification of potential biomarker to separately distinguish patients’ ocular samples of DCAE DCE, DCAE4 and DCE4 groups from the healthy volunteers’ ocular samples of the CON group.** (A) Using Random Forest analysis and the ROC validation, the presented model evaluated the top important genera to distinguish the DCAE patients from the CON before the dacryocystorhinostomy. (B) Before the dacryocystorhinostomy, the top important genera to distinguish the DCE patients from the CON. (C) After the dacryocystorhinostomy, the top important genera to distinguish the DCAE4 patients from the CON. (D) After the dacryocystorhinostomy, the top important genera to distinguish the DCE4 patients from the CON. The X-axis represents the number of variables (genera) of the importance ranking TOPn, and the Y-axis represents the Area Under Curve (AUC) values of receiver operating characteristic curve (ROC) analyses using the corresponding number of variables (genera). The bottom right corner of each panel lists biomarkers that can be used to distinguish patients’ ocular samples of DCAE DCE, DCAE4 and DCE4 groups from the healthy volunteers’ ocular samples of CON group.

**Figure S5 Significantly different genera when comparing each pair of the four time points with and without antibiotic pre-treatment in DC patients.** (A) Significantly different genera of DCAE and DCAE1 groups. (B) Significantly different genera of DCAE and DCAE2 groups. (C) Significantly different genera of DCAE and DCAE4 groups. (D) Significantly different genera of DCE and DCE1 groups. (E) Significantly different genera of DCE and DCE2 groups. (F) Significantly different genera of DCE and DCE4 groups. The Mann–Whitney U test was carried out for the two groups. * FDR < 0.05, ** FDR < 0.01, *** FDR < 0.001.

**Figure S6** **Two ocular microbial types and their distribution in different ocular diseases patients.** (A) Two different ocular microbial types were identified based on the genera from healthy participants and all ocular diseases patients. (B) The microbial genera composition of the two different ocular microbial types based on healthy participants and all ocular diseases patients. Only genera with relative abundance of more than 1% are listed. (C) The prevalence of the two ocular microbial types in healthy participants and in patients with different ocular diseases, including DC, ocular mucosa-associated lymphoid tissue (MALT), stenosis of lacrimal canaliculus (SLC), and chronic dacryocanaliculitis patients (DCC).

**Figure S7 Comparison of ocular and nasal microbiota of DC patients.** (A) Comparison of microbial alpha diversity with Ace index between ocular and nasal microbiota. (B) Comparison of microbial alpha diversity with Shannon index between ocular and nasal microbiota. The Kruskal–Wallis test with Tukey–Kramer post hoc test was employed to test microbial alpha diversity differences. (C) Comparison of microbial beta diversity with the ANOSIM analysis based on Bray–Curtis distance matric between ocular and nasal microbiota. (D) The microbial genera composition of the overall ocular and nasal microbiota from DC patients. Only genera with relative abundance of more than 1% are listed. (E) Significantly different genera when comparing ocular microbiota with nasal microbiota. The Mann–Whitney U test was carried out for the two groups. * FDR < 0.05, ** FDR < 0.01, *** FDR < 0.001.

**Figure S8 Significantly different genera when comparing nasal microbiota from DC patients with (DCAN) and without (DCN) antibiotic pre-treatment, both before and at the fourth week after the dacryocystorhinostomy (DCAN4 and DCN4).** (A) Significantly different genera of DCAN and DCAN4 groups. (B) Significantly different genera of DCN and DCN4 groups. (C) Significantly different genera of DCAN and DCN groups. (D) Significantly different genera of DCAN4 and DCN4 groups. The Mann–Whitney U test was carried out for the two groups. * FDR < 0.05, ** FDR < 0.01, *** FDR < 0.001.
